# Chromosomal integration of the pSOL1 megaplasmid of *Clostridium acetobutylicum* for continuous and stable advanced biofuels production

**DOI:** 10.1038/s41564-024-01714-w

**Published:** 2024-06-14

**Authors:** Muhammad Ehsaan, Minyeong Yoo, Wouter Kuit, Céline Foulquier, Philippe Soucaille, Nigel P. Minton

**Affiliations:** 1 BBSRC/EPSRC Synthetic Biology Research Centre, School of Life Sciences, Biodiscovery Institute, University of Nottingham, Nottingham, UK; 2 TBI, Université de Toulouse, CNRS, INRAE, INSA, Toulouse, France

**Keywords:** Metabolic engineering, Metabolic engineering

## Abstract

Biofuel production by *Clostridium acetobutylicum* is compromised by strain degeneration due to loss of its pSOL1 megaplasmid. Here we used engineering biology to stably integrate pSOL1 into the chromosome together with a synthetic isopropanol pathway. In a membrane bioreactor continuously fed with glucose mineral medium, the final strain produced advanced biofuels, *n*-butanol and isopropanol, at high yield (0.31 g g^−1^), titre (15.4 g l^−1^) and productivity (15.5 g l^−1^ h^−1^) without degeneration.

## Abstract (web summary)

The stable integration of a 192 kb plasmid into the chromosome of *Clostridium acetobutylicum* by a synthetic biology approach enables continuous production of *n*-butanol.

## Main

The Weizmann process for acetone and *n*-butanol production by *Clostridium acetobutylicum* was the second largest fermentation process (after ethanol) and of considerable industrial, social and historical importance^1–3^. Beyond its use during the First World War to produce acetone for smokeless gunpowder (cordite) manufacture, it was used worldwide to produce these two industrial solvents from a variety of renewable substrate^3–5^. Its demise in the early 1960s was a consequence of superior petrochemical-process economics. The fermentation process suffered from low yield, titre and productivity^6^ and, unlike rival petrochemical processes, was not suited to continuous-process technologies due to the loss of *C. acetobutylicum*’s capacity to produce solvents, known as ‘degeneration’. This was associated with loss of the pSOL1 megaplasmid carrying genes essential to solvent production^7,8^. Today, there is a resurgence of interest in *C. acetobutylicum* for the production of advanced fuels (1) after chemical transformation of the solvents mixture^9,10^ or (2) directly as isopropanol–*n*-butanol–ethanol (IBE) mixtures^11,12^.

**Fig. 1:**
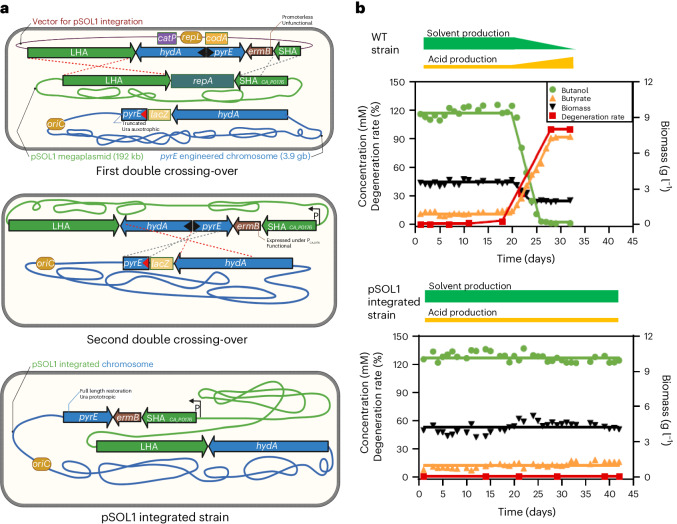
Construction of pSOL1 megaplasmid integrated *C. acetobutylicum* producing solvent stably in continuous culture. **a**, Schematic illustration of pSOL1 integrated strain by using original synthetic approach based on counter selection markers and antibiotic markers selection. Genes in blue boxes are originally located on the chromosome, whereas genes in green boxes are originally located on pSOL1. The black triangle in the gene box presents 5′ truncation, while the red triangle presents 3′ truncation. P below the black arrow indicates the position of the natural promoter of *CA_P0176*. **b**, Product concentration and degeneration rate of wild-type and pSOL1 integrated strains in continuous cultures under the same conditions. The butanol and butyrate concentrations and degeneration rates are plotted on the left *y* axis, with biomass plotted on the right *y* axis. Source data

Here we successfully addressed the issue of strain degeneration in the Weizmann process by using an innovative synthetic biology approach. Our method involved integrating pSOL1 into the chromosome of *C. acetobutylicum* at the *pyrE* locus of an allele-coupled exchange (ACE)^13^ compatible strain with a deletion in the 5′ region of *pyrE* (Fig. 1a).

**Extended Data Fig. 1:**
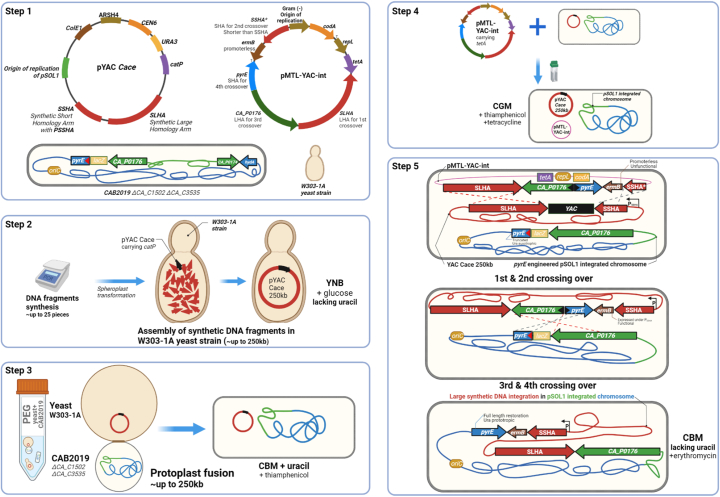
Strategy for integrating large synthetic DNA into the pSOL1 integrated strain. To integrate large, synthetic DNA regions carrying novel metabolic or biosynthetic capabilities for further engineering, the following strategy is proposed. This process involves five steps, including the use of Yeast Artificial Chromosome (YAC) to assemble the large synthetic DNA, protoplast fusion to bring about its transfer into the target cell and final integration using a similar approach as demonstrated in this study. More details are provided below. *Step 1* Preparation of *C. acetobutylicum* and yeast strains and vectors. CAB2019 strain carrying truncated *pyrE* with deletions of *CA_C1502/CA_C3535* genes encoding restriction endonucleases^32^, a yeast strain that is auxotrophic for several nutrients including uracil (*URA3*-deficient), that is W303-1A (genotype: *MAT*a *leu2-3,112 his3-11,15 trpl-1 canl-100 ade2-7 ura3-1*)^33,34^ as well as the 2 following plasmids: i) pYAC Cace, YAC derivative containing 1) a yeast origin of replication (*ARSH4*), a centromere (*CEN6*), an uracil-selectable marker (*URA3*), 2) the origin of replication of pSOL1 and a *catP* gene and 3) synthetic SLHA and SSHA regions for the crossing over events. ii) pMTL-YAC-int, a derivative of pMTL-pSOL1 int, carrying *tetA* (replacing *catP*), *CA_P0176* (replacing *hydA* to be LHA) and the SSHA’ (shorter than SHA) and SLHA that are homologous regions of pYAC Cace vector. *Step 2* Synthetic DNA assembly using pYAC Cace as a backbone. Up to 25 fragments can be synthesized and transformed into yeast^35,36^. A large DNA vector including pYAC Cace backbone is assembled in W303-1A yeast strain to generate lengths of up to 250 kb. Commercial Yeast Nitrogen Base (YNB) with all essential except uracil is used for selection after complementing with *URA3* on the pYAC Cace vector. Additionally, the resultant plasmid pYAC Cace 250 kb carries the *catP* antibiotic resistance marker for step 3 selection. *Step 3* Protoplast fusion between the yeast carrying pYAC Cace 250 kb and CAB2019 by treating PEG (Polyethylene glycol) to induce fusion. PEG treated *C. acetobutylicum*^37^ and yeast for protoplast induction are mixed. After protoplast fusion, CBM supplemented with uracil (to select *C. acetobutylicum* by deficiency of amino acids such as histidine due to auxotrophy of W303-1A) and thiamphenicol (to maintain pYAC Cace 250 kb) is used for selection. *Step 4* Introduction of pMTL-YAC-int into CAB2019 harbouring pYAC Cace 250 kb by electroporation. Transformant is selected on CGM supplemented with tetracycline (*tetA* in pMTL-YAC-int) and thiamphenicol. *Step 5* Four crossing over events to integrate 250 kb large DNA into pSOL1 integrated *C. acetobutylicum*. First and second crossing over occurs between SLHAs (Synthetic Large Homology Arms)/SSHA (Synthetic Short Homology Arm) and SSHA’ of pYAC Cace 250 kb and pMTL-YAC-int. The promoter-less *ermB* gene from pMTL-YAC-int becomes functional after integration as it is now located under the promoter of SSHA in pYAC Cace 250 kb while thiamphenicol sensitivity is expected due to loss of pYAC backbone by use of 5FC. Third and fourth crossing over occurs between *CA_P0176* and *pyrE* of pSOL1 integrated chromosome and the resultant plasmid after pYAC Cace 250 kb and pMTL-YAC-int integration. As the truncated *pyrE* is restored to full length after the 4^th^ crossing over, the final transformant can grow on CBM without uracil supplementation.

This strategy involves four crossing-over events and the use of an ACE replicative plasmid^13^ introduced by electroporation (Supplementary Note 1) and containing the following: (1) homologous regions flanking the origin of replication of pSOL1 (*repA*), (2) a truncated *pyrE-hydA* locus in reverse orientation with a full-length but promoterless *ermB* gene that becomes functional only after the initial crossing-over of SHA (*CA_P0176*) with pSOL1 and (3) a functional *codA* gene. The first double crossing-over occurs between the two homologous regions surrounding the origin of replication of pSOL1 also present on the ACE plasmid. Each homology arm size was specifically designed to control the integration order. As a result, the engineered strain contains a pSOL1 plasmid lacking an origin of replication but having a functional *ermB* now expressed under the natural *CA_P0176* promoter on pSOL1. However, this pSOL1 intermediate cannot replicate and is therefore unstable. Consequently, a second double crossing-over event at the *pyrE* locus is necessary to ensure its maintenance through integration (Fig. 1a), as the mutant having pSOL1 integration is selected on a uracil-lacking *Clostridium* basal medium (CBM) requiring functional *pyrE* restoration. Thus, mutants having a clean integration of pSOL1 after four crossing-over events (Fig. 1a) were directly obtained by spreading 10^9^ of ACE plasmid containing cells on a CBM agar lacking uracil and supplemented with erythromycin. Colonies obtained were then replica plated on CBM agar containing erythromycin and 5-fluorocytosine (5FC) (Supplementary Note 2). Resistance to 5FC confirms that all the clones were cured of the original ACE plasmid, as well as the hybrid ACE plasmid with two origins of replication that was generated after the first double crossing-over event. The accurate genotype and phenotype of the mutants were validated through PCR and growth in batch cultures. The resultant CAB2018 strain showed the correct PCR and product profiles in batch culture, as well as an equivalent sporulation phenotype to the wild-type strain (Supplementary Note 4). Its specific growth rate, however, was around 30% lower than that of the wild-type strain (Supplementary Note 4). Although we have no explanation for this phenotype, it could explain why pSOL1 did not integrate evolutionarily into the chromosome of *C. acetobutylicum*. Whole-genome sequencing of the strain confirmed that pSOL1 had integrated as expected and that four single-nucleotide polymorphisms in non-relevant chromosomal genes had been acquired (Supplementary Table 2). To the best of our knowledge, we have not found any study reporting the stable integration of such a big piece of DNA into the chromosome of any bacteria. In Extended Data Fig. 1, we proposed a similar strategy for the integration of any large pieces of DNA into the chromosome of the CAB2019 strain, a CAB2018-derived strain with *pyrE* truncated and *ermB* removed (Supplementary Note 3) using the following: (1) an engineered yeast artificial chromosome (YAC) vector with the origin of replication of pSOL1, (2) a yeast strain to assemble the large synthetic DNA insert and (3) protoplast fusion to introduce the large vector into the CAB2019 strain. This extension of the method will be of particular value when the introduction of complex metabolic pathways comprising numerous genes is required, for example, the Wood–Ljungdahl pathway for autotrophic growth^14^. Finally, from a fundamental point of view, one of the benefits of having pSOL1 integration into the chromosome is that it now allows the rapid functional analysis of the pSOL1-encoded genes using CRISPR–Cas9 for gene editing^15^.

Monitoring of continuous cultures of the wild-type and CAB2018 strains showed that whereas the former degenerated within 30 days, losing the ability to produce solvents concomitant with loss of pSOL1, the latter strain remained stable over the 42 day fermentation period (Fig. 1b). Having a stable strain that produces *n*-butanol in continuous cultures is a prerequisite, but alone is not sufficient, for a commercial biofuel production process as both the yields, titres and productivities are too low due to production of acetone, a natural final product that is not suitable as a biofuel.

**Fig. 2:**
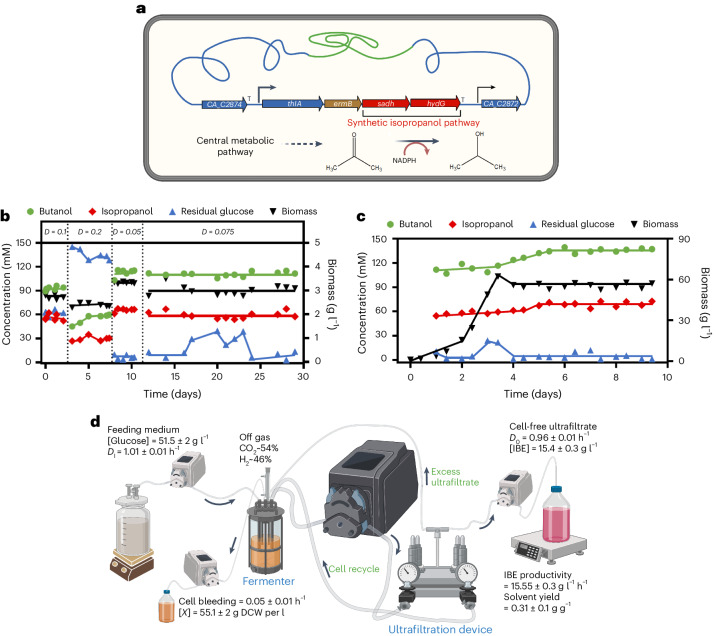
Synthetic biofuel pathway integration into pSOL1 integrated strain and stable solvent production in GMM in continuous cultures of CAB2021 strain. **a**, *sadh* (encoding an NADPH)-dependent acetone reductase) and *hydG* (encoding putative ferredoxin-NADP^+^ reductase) of *C. beijerinckii* NRRL B593 for conversion of acetone to isopropanol were integrated downstream of *thlA* to be under the strong *thlA* promoter in CAB2021. **b**, Isopropanol, butanol, productivity and yield of four different dilution rates in continuous chemostat culture of CAB2021. Biofuel is defined as (isopropanol + butanol + ethanol). *D*, dilution rate (h^−1^). **c**, Continuous production of advanced biofuel by CAB2021 in membrane cell-recycle bioreactor. Same symbols are used as in **b**. **d**, Membrane cell-recycle bioreactor used in this study. Data are shown as mean and s.d. of steady state period (day 4 to day 10, *n* = 12). DCW, dry cell weight (g); *D*_i_, incoming dilution rate (h^−1^); *D*_o_, outcoming dilution rate (h^−1^). Source data

**Extended Data Fig. 2:**
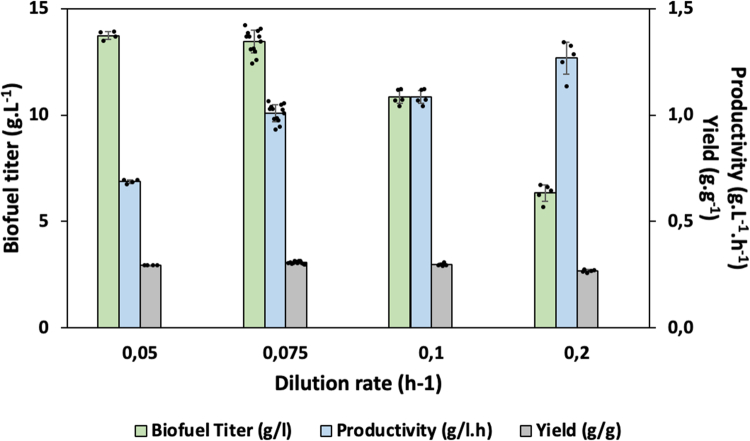
Biofuel titre, productivity, and yield of CAB2021 strain grown in chemostat culture in GMM at different dilution rates. To determine the value of CAB2021 for a commercial biofuel production process, an economical mineral medium GMM was continuously fed and different dilution rates were tested. In terms of biofuel titre, the dilution rates of 0.05 and 0.075 h^−1^ showed the highest titre. In terms of productivity, a dilution rate of 0.2 h^−1^ showed the highest productivity while high residual glucose was seen (Fig. 2b). In terms of yield, no significant difference was seen among all the tested dilution rates. Data are shown as mean ± SD from biological replicates (a replicate number for each dilution rate is as following, dilution rate 0.05 h^−1^, n = 5; 0.075 h^−1^, n = 13; 0.1 h^−1^, n = 6; 0.2 h^−1^, n = 6). Source data

Accordingly, to improve the strain for continuous biofuels production, we further engineered the strain to continuously produce an advanced IBE fuel mixture at high practical yield and productivity. This was achieved in three steps involving two intermediate strains (CAB2019 and CAB2020) and the final CAB2021strain with integration of *sadh* and *hydG* genes (Fig. 2a and Supplementary Note 3) of *Clostridium beijerinckii* NRRL B593^16^ under the control of the natural *thlA* promoter. These encode a reduced nicotinamide adenine dinucleotide phosphate (NADPH)-dependent acetone reductase (*sadh*) and a potential ferredoxin-NADP^+^ reductase (*hydG*). In batch culture, the CAB2021 strain almost completely converted acetone to isopropanol (Supplementary Note 5). Furthermore, when grown in chemostat cultures at different dilution rates on a glucose mineral media (GMM), it was possible to produce biofuels at high yield (0.3 g g^−1^) and productivity (1.3 g l^−1^ h^−1^) for the highest dilution rate evaluated, although under this condition high residual glucose concentrations were observed (Fig. 2b and Extended Data Fig. 2). The best compromise between residual glucose, titre and productivity was observed at a dilution rate of 0.075 h^−1^ with a biofuel titre of 13.4 g l^−1^, a productivity of 1 g l^−1^ h^−1^ and a yield of 0.31 g g^−1^. These values were much higher than those obtained with *C. beijerinckii* NRRL B593, the natural producer, or a metabolically engineered mutant of *C. acetobutylicum* expressing *sadh* inserted at the *pyrE* locus^17^, when grown in chemostat cultures (Supplementary Table 4). Furthermore, contrary to the natural producer that loses its ability to produce solvent after 10 to 20 days in continuous cultures^18^, no instability was observed with CAB2021 for at least 30 days (Fig. 2b). Finally, to evaluate the potential of strain CAB2021 for industrial production of advanced biofuels, we used it in a membrane cell-recycle bioreactor system (Fig. 2c,d) to increase the cell density and then the productivities^19^. In GMM, the performances obtained in terms of yield (0.31 g g^−1^), titre (15.4 g l^−1^) and productivities (15.5 g l^−1^ h^−1^) were the highest ever reported for the continuous production of an advanced IBE fuel mixture^17,19–25^ (Fig. 2d and Supplementary Table 4). We recently used an advanced extractive fermentation process to improve the performances of a metabolically engineered *C. acetobutylicum* optimised for *n*-butanol production at high yield^26^. This process, using vacuum distillation and high cell density culture, could be used with the CAB2021 strain developed here to further increase the titre of the advanced biofuel mixture produced.

In summary, engineering biology was used to develop a *C. acetobutylicum* strain for the stable and continuous production of advanced biofuels from a low-cost GMM at higher yield and productivities than previously reported. To further develop an economical industrial process, performances still need to be improved. This might be achievable by the following: (1) further metabolic engineering to remove by-product pathway such as butyrate^11,27^ to increase the biofuels yield and/or (2) optimisation of the bioreactor by using extractive fermentation^26^.

## Methods

### Reagents

All chemicals used in this study were purchased from Sigma-Aldrich unless otherwise noted. Q5 High-Fidelity DNA Polymerase (NEB) or KOD DNA Polymerase (Merck) was used for PCR. All PCR products and plasmids were purified and extracted with the PCR purification kit and plasmid extraction kit (Qiagen), respectively. All oligonucleotides were purchased from Sigma-Aldrich or Integrated DNA Technologies. DNA sequencing was performed at Eurofins. Genomic DNA of CAB2018 strain was isolated by phenol:chloroform extraction based on the method of Marmur^28^. After measuring (1) the amount of genomic DNA with a NanoDrop lite spectrophotometer (Thermo Scientific) and (2) its quality via agarose gel electrophoresis, whole-genome sequencing was performed using an Illumina MiSeq benchtop sequencer (Deepseq, University of Nottingham). Sequence reads were mapped to the reference sequences NC_003030 (chromosome) and NC_001988 (pSOL1) in the NCBI database using the program CLC Genomics Workbench version 22.0.1 (Qiagen).

### Bacterial strains and medium composition

Bacterial strains and plasmids used in this study are detailed in Supplementary Table 1. *Escherichia coli* TOP10 (Invitrogen) was cultured aerobically (37 °C; shaking at 200 r.p.m.) in Luria–Bertani medium supplemented with chloramphenicol (25 μg ml^−1^) and tetracycline (10 μg ml^−1^) where appropriate for plasmid cloning and propagation, and TOP10 containing pAN2 plasmid was used for in vivo methylation of plasmid DNA before transformation of *C. acetobutylicum* ATCC 824 and any other recombinant strains (Supplementary note 1). *C. acetobutylicum* strains were routinely grown anaerobically at 37 °C using pre-reduced overnight CBM or Clostridium Growth Medium (CGM) agar supplemented with thiamphenicol (15 µg ml^−1^) or erythromycin (20 μg ml^−1^) where appropriate under an atmosphere of N_2_:H_2_:CO_2_ (80:10:10, vol:vol:vol) in an anaerobic workstation (Don Whitley) or in anoxic broth in a serum bottle. Recovery after transformation was carried out in anoxic 2×YTG (pH 5.2) broth in the anaerobic workstation. Uracil was supplemented at 20 µg ml^−1^ when needed. 5FC was supplemented at a final concentration of 100 µg ml^−1^ where *codA*-based selection was performed. *C. acetobutylicum* strain degeneration was evaluated in chemostat culture in *Clostridium* Rich Media (CRM). Performances of the engineered *C. acetobutylicum* strains both in chemostat and in a membrane cell-recycle bioreactor were evaluated in GMM.

The CBM contains (per litre) 50 g glucose, 0.5 g K_2_HPO_4_, 0.5 g KH_2_PO_4_, 0.2 g MgSO_4_·7H_2_O, 7.58 mg MnSO_4_·H_2_O, 0.01 g FeSO_4_·7H_2_O, 1 mg para-aminobenzoic acid (PABA), 0.002 mg biotin, 1 mg thiamine HCl, 4 g casein hydrolysate and 5 g CaCO_3_ if needed as buffering agent. The CGM contains (per litre) 0.75 g KH_2_PO_4_, 0.75 g K_2_HPO_4_, 0.4 g MgSO_4_·7H_2_O, 0.01 g MnSO_4_·H_2_O, 0.01 g FeSO_4_·7H_2_O, 1 g NaCl, 2 g asparagine, 5 g yeast extract, 2 g (NH4)_2_SO_4_ and 60 g glucose. The 2×YTG medium contains (per litre) 16 g tryptone, 10 g yeast extract, 5 g NaCl and 10 g glucose. The CRM contains (per litre) 50 g glucose, 4 g yeast extract, 0.5 g K_2_HPO_4_, 0.5 g KH_2_PO_4_, 0.2 g MgSO_4_·7H_2_O, 10 mg NaCl, 10 mg FeSO_4_·7H_2_O, 10 mg MnSO_4_·H_2_O, 80 μg biotin and 8 mg PABA. The GMM medium contains (per litre) 50 g glucose, 0.5 g K_2_HPO_4_, 0.5 g KH_2_PO_4_, 1.5 g NH_4_Cl, 0.2 g MgSO_4_·7H_2_O, 10 mg FeSO_4_·7H_2_O, 80 μg biotin and 8 mg PABA.

### Plasmids construction

Plasmid pMTL-pSOL1-int (Fig. 1, Supplementary Table 1 and Supplementary Fig. 1) was constructed using plasmid pMTL-SC7515 carrying *codA*, *repL* and *catP*^29^ as a backbone. This vector was designed to contain four different sizes of homology arms: (1) 1,200 bp of *CA_P0173-4* homology arm (LHA, long homology arm for first crossing-over with the pSOL1), (2) 305 bp of *CA_P0176* homology arm (SHA, short homology arm for second crossing-over with pSOL1 to excise replication origin *repL* encoded by *CA_P0175* of pSOL1 and to introduce 3′ truncated *pyrE* and 3′ truncated *hydA* into pSOL1), (3) 900 bp of *hydA* homology arm (LHA for third crossing-over with chromosome) and (4) 635 bp of *pyrE* homology arm (40 bp from start codon truncated SHA for fourth crossing-over with 3′ truncated *pyrE* carrying chromosome to restore full-length functional *pyrE* at the last step). As previously reported^13^, the use of asymmetrical homology arm sizes can control the order of recombination events, allowing the initial isolation of single crossing-over integrants involving the LHAs followed by the selection of recombinants arising from subsequent, double crossing-over, excision events involving the SHAs. The cassette that consists of four homology arms and promoterless *ermB* gene was synthesized (Biomatik) in the order of a 1,200 bp LHA *CA_P0173-4* for pSOL1 integration, terminator, reverse-oriented 900 bp *hydA* LHA for chromosome integration, reverse-oriented 635 bp of *pyrE* SHA for chromosome integration, terminator, *ermB* with its ribosome binding site but without promoter to be active after integration and 305 bp of *CA_P0176* SHA. This cassette was cloned at the PmeI site of pMTL-SC7515 to construct pMTL-pSOL1-int. Plasmid pMTL-pSOL1-int was used to construct the CAB2018 strain.

Plasmid pMTL-pSOL1-*pyrE*-Negative was constructed to remove (1) the erythromycin resistance gene *ermB* and (2) 335 bp of the *pyrE* gene of CAB2018 by double crossing-over.

Previously constructed plasmid pMTL-JH12^13^, which was made for *pyrE* 5′ truncation, was used as a backbone. The LHA (*hydA* fragment in pMTL-JH12) was replaced by the PCR fragment for *CA_P0176-77* homology by using primers Pr1 and Pr2, whereas the remaining part, including the SHA (partial *pyrE* for truncation), *lacZ* (MCS) and the antibiotic resistance marker *catP* in the original plasmid was retained. DNA fragment replacement was carried by enzyme digestions with NheI/AscI (NEB) and ligation with T4 DNA ligase (NEB). Plasmid pMTL-pSOL1-*pyrE*-Negative was used to construct the CAB2019 strain.

Plasmid pMTL-pSOL1-*pyrE*-Positive was constructed to restore the deleted 335 bp *pyrE* fragment in CAB2019 for a full-length functional *pyrE*.

To restore functional *pyrE*, a synthetic DNA fragment for full-length *pyrE*—a Rho-independent terminator—*CA_P0176-77* ended with SbfI/AscI (Biomatak) was cloned into SbfI/AscI (NEB) digested pMTL-pSOL1-*pyrE*-Negative plasmid to replace partial *pyrE-lacZ* fragment. Plasmid pMTL-pSOL1-*pyrE*-Positive was used to construct the CAB2020 strain.

Plasmid pMTL-JH16-*sadh-hydG* B593 was constructed to integrate a synthetic isopropanol production pathway into the CAB2020 strain for stable and continuous production of advanced biofuels. It carries part of *thlA* (thiolase encoding gene, left SHA for recombination to maintain strong expression of inserted genes under *thlA* promoter throughout growth), *ermB* (erythromycin, antibiotic resistance marker for double crossing-over selection) and heterologous *sadh* (encoding a primary/secondary alcohol dehydrogenase) and *hydG* (encoding a putative electron transfer protein) genes from *C. beijerinckii* NRRL B593. The *sadh-hydG* DNA fragment, amplified by using primers Pr3 and Pr4, was cloned into NotI/NheI (NEB, UK) digested plasmid pMTL-JH16^13^. Plasmid pMTL-JH16-*sadh-hydG* B593 was used to construct the CAB2021 strain.

### Cultivation conditions

All liquid cultures of *C. acetobutylicum* strains were performed in 30 or 60 ml serum bottles under strict anaerobic conditions in CGM or GMM in which glucose concentration was 60 g l^−1^ and NH_4_Cl was replaced by ammonium acetate at 2.2 g l^−1^. All serum bottle batch culture data are shown as mean ± s.d. from biological replicates (*n* = 3). Spores were germinated by immersing the serum bottles in a water bath at 80 °C for 15 min.

### Bioreactor cultivation

All chemostat cultures were carried out under strict anaerobic conditions in a 500 ml jacketed bioreactor (BBI-Biotech) with a working volume of 300 ml, an agitation speed set at 200 r.p.m. and pH controlled by automatic addition of NH_4_OH (5 N). Chemostat cultures, in CRM, at 35 °C, a dilution rate of 0.05 h^−1^ and a pH of 4.8, were used to evaluate the strains’ degeneration as it was previously shown that *C. acetobutylicum* continuous cultures were unstable under these conditions^30^. Performances of the CAB2021 strain both in chemostat and in a membrane cell-recycle bioreactor were evaluated in GMM at a pH of 4.4 and a temperature of 35 °C as it was previously shown to give the highest yield of solvent formation^31^. CAB2021 chemostat culture was carried out in multi-stage mode to test different dilution rates. Stage 1 was maintained at a dilution rate of 0.1 h^−1^ for 2 days, and after confirming stable metabolite patterns for 6 time points, the dilution rate was increased to 0.2 h^−1^ to enter stage 2. After confirming high concentration of residual glucose for 6 time points, the dilution rate was decreased to 0.05 h^−1^ to enter stage 3. After confirming stable metabolite patterns and low concentration of residual glucose for 5 time points, the dilution rate was increased to 0.75 h^−1^ to enter stage 4. This stage was run for 13 time points. Continuous cultures in the cell recycle membrane bioreactor were carried out in a 500 ml bespoke glass bioreactor connected to an ultra-filtration flat module (INSIDE KeRAM, TAMI Industries, molecular weight cut-off 50 kDa, surface area 0.25 m^2^). The fermentation broth was recirculated in the membrane by a peristaltic pump (Masterflex 77965-00, Fisher Scientific). The total working volume was 450 ml, and the pH and the temperature were respectively regulated at 4.4 (with 5 N NH_4_OH) and 35 °C. After sterilisation, N_2_ was sparged in the reactor. After a 10% inoculation, the cell recycle membrane bioreactor was operated in batch mode for 9 h before switching to continuous mode with total cell recycle and a step-by-step increase in dilution rate of permeate from 0.04 to 0.96 h^−1^. When an optical density at 600 nm (OD_600nm_) of 200 was reached, a cell bleeding rate was applied at a dilution rate of 0.05 h^−1^.

### Microscope image

Culture broth (1 ml) was centrifuged at 13,000 *g* for 1 min, and the supernatant was discarded. The cells were washed with 1 ml deionized water and centrifuged at 13,000 *g* for 1 min, and 950 μl of supernatant was discarded. Pellets and the remaining supernatant were mixed by pipetting, and 10 μl of the mixture was spotted on a glass microscope slide and covered with a 0.17 mm microscope cover. Immersion oil (10 μl) for microscopy (Merck) was spotted onto the microscope cover glass. Microscopic images were obtained using a Nikon OPTIPHOT-2 microscope at ×1,000 magnification.

### Analytical procedures

Biomass concentration was monitored both by OD_600nm_ using a spectrophotometer and by the dry cell weight method after centrifugation of 1.5 ml of culture broth in an Eppendorf tube (16,000 *g*, 5 min, room temperature), two washes with Milli-Q water and drying under vacuum at 80 °C. The concentrations of glucose, glycerol, acetate, butyrate, lactate, pyruvate, acetoin, acetone, ethanol, isopropanol and *n*-butanol were determined based on high-performance liquid chromatography (HPLC) using H_2_SO_4_ at 0.5 mM as mobile phase. Metabolite concentrations were determined by HPLC analysis (Dionex UltiMate 3000 HPLC system, Thermo Fisher Scientific). Biorad Aminex HPX-87H column (300 mm × 7.8 mm) was used for separation, and a refractive index detector (to analyse glucose, ethanol, butanol and isopropanol) and UV detectors (absorbance at 210 nm for acids, 280 nm for acetone) were used for detection, respectively. Samples were diluted two times and run at a flow rate of 0.5 ml min^−1^ at 35 °C (if applicable, 20 °C was used to better resolve peaks of acetone and isopropanol) in 5 mM H_2_SO_4_ mobile phase for an hour.

### Reporting summary

Further information on research design is available in the Nature Portfolio Reporting Summary linked to this article.

### Supplementary information

Supplementary Information Ten supplementary notes, 12 supplementary figures and four supplementary tables are included in one supplementary information file to support this study.

Reporting Summary

Supplementary Data 1 Assembly of whole-genome sequencing data of CAB2018 strain.

### Source data

Source Data Fig. 1 Statistical source data.

Source Data Fig. 2 Statistical source data.

Source Data Extended Data Fig. 2 Statistical source data.

## Data Availability

Data supporting the work are available in the paper and Supplementary Information. The raw whole-genome sequencing data of CAB2018 are available in the European Nucleotide Archive with accession number ERR12915945, and the assembly file in GenBank format is provided as supplementary data. Further information on materials of this study are available from the corresponding author. Source data are provided with this paper.

## References

[1] Jones DT, Woods DR (1986). Acetone–butanol fermentation revisited. Microbiol. Rev..

[2] Weizmann, C. Improvements in the bacterialfFermentation of carbohydrates and in bacterial cultures for the same, GB patent 191504845A (1915).

[3] Weizmann, C. Production of acetone and alcohol by bacteriological process, US patent 1315585A (1919).

[4] Ndaba B, Chiyanzu I, Marx S (2015). *n*-Butanol derived from biochemical and chemical routes: a review. Biotechnol. Rep..

[5] Tracy BP, Jones SW, Fast AG, Indurthi DC, Papoutsakis ET (2012). Clostridia: the importance of their exceptional substrate and metabolite diversity for biofuel and biorefinery applications. Curr. Opin. Biotech..

[6] Dodds DR, Gross RA (2007). Chemicals from biomass. Science.

[7] Cornillot E, Soucaille P (1996). Solvent-forming genes in clostridia. Nature.

[8] Cornillot E, Nair RV, Papoutsakis ET, Soucaille P (1997). The genes for butanol and acetone formation in *Clostridium acetobutylicum* ATCC 824 reside on a large plasmid whose loss leads to degeneration of the strain. J. Bacteriol..

[9] Anbarasan P (2012). Integration of chemical catalysis with extractive fermentation to produce fuels. Nature.

[10] Bormann S (2014). Engineering *Clostridium acetobutylicum* for production of kerosene and diesel blendstock precursors. Metab. Eng..

[11] Dusseaux S, Croux C, Soucaille P, Meynial-Salles I (2013). Metabolic engineering of *Clostridium acetobutylicum* ATCC 824 for the high-yield production of a biofuel composed of an isopropanol/butanol/ethanol mixture. Metab. Eng..

[12] Papoutsakis ET (2015). Reassessing the progress in the production of advanced biofuels in the current competitive environment and beyond: what are the successes and where progress eludes us and why. Ind. Eng. Chem. Res..

[13] Heap JT (2012). Integration of DNA into bacterial chromosomes from plasmids without a counter-selection marker. Nucleic Acids Res..

[14] Köpke M (2010). *Clostridium ljungdahlii* represents a microbial production platform based on syngas. Proc. Natl. Acad. Sci. USA.

[15] Wilding-Steele, T., Ramette, Q., Jacottin, P. & Soucaille, P. Improved CRISPR/Cas9 tools for the rapid metabolic engineering of *Clostridium acetobutylicum*. *Int. J. Mol. Sci.* 22, (2021). 10.3390/ijms22073704 PMC8037352 33918190

[16] Jang YS (2013). Metabolic engineering of *Clostridium acetobutylicum* for the enhanced production of isopropanol–butanol–ethanol fuel mixture. Biotechnol. Prog..

[17] Bankar SB, Jurgens G, Survase SA, Ojamo H, Granstrom T (2015). Genetic engineering of *Clostridium acetobutylicum* to enhance isopropanol–butanol–ethanol production with an integrated DNA-technology approach. Renew. Energy.

[18] Jobses IML, Roels JA (1983). Experience with solvent production by *Clostridium beijerinckii* in continuous culture. Biotechnol. Bioeng..

[19] Survase SA (2019). Membrane assisted continuous production of solvents with integrated solvent removal using liquid–liquid extraction. Bioresour. Technol..

[20] Ahmed I, Ross R, Mathur V, Chesbro W (1988). Growth rate dependence of solventogenesis and solvents produced by *Clostridium beijerinckii*. Appl. Microbiol. Biotechnol..

[21] Survase SA, Jurgens G, van Heiningen A, Granstrom T (2011). Continuous production of isopropanol and butanol using *Clostridium beijerinckii* DSM 6423. Appl. Microbiol. Biotechnol..

[22] Carrié M, Velly H, Ben-Chaabane F, Gabelle JC (2022). Modeling fixed bed bioreactors for isopropanol and butanol production using DSM 6423 immobilized on polyurethane foams. Biochem. Eng. J..

[23] Yang Y, Hoogewind A, Moon YH, Day D (2016). Production of butanol and isopropanol with an immobilized *Clostridium*. Bioproc. Biosyst. Eng..

[24] Krouwel P, Groot W, Kossen N (1983). Continuos IBE fermentation by immobilized growing *Clostridium beijerinckii* cells in a stirred‐tank fermentor. Biotechnol. Bioeng..

[25] Groot W, Van der Lans R, Luyben KCA (1989). Batch and continuous butanol fermentations with free cells: integration with product recovery by gas-stripping. Appl. Microbiol. Biotechnol..

[26] Nguyen NP, Raynaud C, Meynial-Salles I, Soucaille P (2018). Reviving the Weizmann process for commercial *n*-butanol production. Nat. Commun..

[27] Yoo M, Croux C, Meynial-Salles I, Soucaille P (2017). Metabolic flexibility of a butyrate pathway mutant of *Clostridium acetobutylicum*. Metab. Eng..

[28] Marmur J (1961). A procedure for the isolation of deoxyribonucleic acid from micro-organisms. J. Mol. Biol..

[29] Ehsaan M (2016). Mutant generation by allelic exchange and genome resequencing of the biobutanol organism *Clostridium acetobutylicum* ATCC 824. Biotechnol. Biofuels.

[30] Ferras E, Minier M, Goma G (1986). Acetonobutylic fermentation—improvement of performances by coupling continuous fermentation and ultrafiltration. Biotechnol. Bioeng..

[31] Yoo M (2015). A quantitative system-scale characterization of the metabolism of *Clostridium acetobutylicum*. mBio.

[32] Croux C (2016). Construction of a restriction-less, marker-less mutant useful for functional genomic and metabolic engineering of the biofuel producer. Biotechnol. Biofuels.

[33] Thomas BJ, Rothstein R (1989). Elevated recombination rates in transcriptionally active DNA. Cell.

[34] Replogle K, Hovland L, Rivier DH (1999). Designer deletion and prototrophic strains derived from strain W303-1a. Yeast.

[35] Gibson DG (2008). Complete chemical synthesis, assembly, and cloning of a genome. Science.

[36] Gibson DG (2008). One-step assembly in yeast of 25 overlapping DNA fragments to form a complete synthetic genome. Proc. Natl Acad. Sci. USA.

[37] Gao XF, Zhao H, Zhang GH, He KZ, Jin YL (2012). Genome shuffling of CICC 8012 for improved production of acetone–butanol–ethanol (ABE). Curr. Microbiol..

