# Supplementary material for: Chromosomal integration of the pSOL1 megaplasmid of Clostridium acetobutylicum for continuous and stable advanced biofuels production
Source: Nat Microbiol. 2024 Jun 14;9(7):1655–60. doi: 10.1038/s41564-024-01714-w (PMC11222136; doi:10.1038/s41564-024-01714-w)
Supplement: Supplementary file 1 — Ten supplementary notes, 12 supplementary figures and four supplementary tables are included in one supplementary information file to support this study. [file 41564_2024_1714_MOESM1_ESM.pdf]

nature microbiology

Supplementary information https://doi.org/10.1038/s41564-024-01714-w

Chromosomal integration of the pSOL1 megaplasmid of *Clostridium acetobutylicum* for continuous and stable advanced biofuels production

In the format provided by the authors and unedited

Table-of-contents

List of Abbreviations

Supplementary Table 1. All bacterial strains, plasmids, and primers used in this study

Supplementary Table 2. List of mutations identified in CAB2018 strain

Supplementary Note 1 | Transformation procedure

Supplementary Note 2 | Construction of CAB2018 strain

  Supplementary Figure 1. Physical map of pMTL-pSOL1-int

  Supplementary Figure 2. pSOL1 integration verification by PCRs in CAB2018

Supplementary Note 3 | Construction of CAB2019, CAB2020 and CAB2021 strains

  Supplementary Figure 3. PCR validation of CAB2019 strain

  Supplementary Figure 4. PCR validation of CAB2020 strain

  Supplementary Figure 5. PCR validation of CAB2021 strain

Supplementary Note 4 | Specific growth rate, products profile in serum bottles and sporulation ability of the wild type and CAB2018 strains

  Supplementary Table 3. Specific growth rate in serum bottles of the wild type and CAB2018 strains

  Supplementary Figure 6. Products profile in serum bottles of the wild type and CAB2018 strains

  Supplementary Figure 7. Sporulation of CAB2018

Supplementary Note 5 | Products profile in serum bottles of the wild type, CAB2020 and CAB2021 strains

  Supplementary Figure 8. Products profile in serum bottles of the wild type and CAB2020 strains

  Supplementary Figure 9. Products profile in serum bottles of the wild type and CAB2021 strains

Supplementary Note 6 | Analysis of pSOL1 loss in wild type and CAB2019 strains

  Supplementary Figure 10. Starch based assay to analyze loss of pSOL1

Supplementary Note 7 | Genome Sequencing of CAB2018 strain

Supplementary Note 8 Comparison of the IBE production by the natural or engineered Clostridia in continuous culture

  Supplementary Table 4 | Comparison of the IBE production by Clostridia in continuous culture

Supplementary Note 9 | Schematic representation of the natural organization of the genes used for engineering in this study

Supplementary Figure 11. Natural organization of the genes used for engineering in this study

Supplementary Note 10 | Central metabolism of *Clostridium acetobutylicum*

Supplementary Figure 12. Simplified central metabolism of *Clostridium acetobutylicum*

List of Abbreviations

ACE Allele-Coupled Exchange

AA Amino Acid

LHA Long Homology Arm

SHA Short Homology Arm

SLHA Synthetic LHA

SSHA Synthetic SHA

GMM Glucose Mineral Medium

CGM Clostridial Growth Medium

CBM Clostridial Basal Medium

Erm Erythromycin

Tm Thiamphenicol

5FC 5-fluorocytosine

FOA 5-Fluoroorotic acid

SD Standard Deviation

YAC Yeast Artificial Chromosome

PEG Polyethylene glycol

YNB Yeast Nitrogen Base

Supplementary Table 1. All bacterial strains, plasmids, and primers used in this study

Strain, plasmid and primer	Relevant characteristics	Source
**Strain**		
E. coli		
pAN2 in Top10	In vivo methylation strain	[1] [2]
Clostridium acetobutylicum		
pyrE engineered	ACE compatible *Clostridium acetobutylicum* strain having a deletion in the 5′ region of *pyrE*	[3]
CAB2018	Entire pSOL1 megaplasmid integrated into chromosome of *C. acetobutylicum* at *pyrE* locus, *ermB* carried	This study
CAB2019	Derived from CAB2018, pSOL1 integrated, *pyrE* deleted, *ermB* removed	This study
CAB2020	Derived from CAB2019, pSOL1 integrated, full-length *pyrE* restored strain, no antibiotic resistance marker carried	This study
CAB2021	Derived from CAB2020, *sadh-hydG* of *C. beijerinckii* integrated at downstream of *thlA* (*CA_C2873*), strong expression intended under *thlA* promoter, *ermB* carried	This study
**Plasmid**		
pMTL-pSOL1 integration	pMTL-SC7515[4] was used as a backbone and a synthetic cassette including 4 homology arms and *ermB* gene was cloned at the PmeI site. Designed to construct pSOL1 integrated strain, CAB2018 strain	This study
pMTL-pSOL1-*pyrE*-Negative	pMTL-JH12[3] derived with *catP*, to remove erythromycin resistance gene and initial 300 bp of *pyrE*. Designed to construct CAB2019 strain	This study
pMTL-pSOL1-*pyrE*-Positive	pMTL-JH12 derived with *catP*, to restore deleted 300 bp *pyrE* of the *pyrE* Negative strain. Designed to construct CAB2020 strain	This study
pMTL-JH16-*sadh-hydG* B593	pMTL-JH12 derived with *catP*, to integrate *ermB* lacking promoter and *sadh/hydG* from *C. beijerinckii* NRRL B593 for synthetic isopropanol pathway under *thlA* promoter by homologous recombination (asymmetrical homology arms to control recombination order at *thlA* lastly). Designed to construct CAB2021 strain	This study

**Primer** [A)]		**Note**
Pr1	aaaagctagcttattattcaaaatctatattattcacgc	To amplify *CA_P0176-77* LHA and replace original fragment with NheI/AscI of JH12 to construct pMTL-pSOL1-*pyrE*-Negative, NheI site, forward
Pr2	aaaaggcgcgccaatgtaataagtataatcaacgttaaag	To amplify *CA_P0176-77* LHA and replace original fragment with NheI/AscI of JH12 to construct pMTL-pSOL1-*pyrE*-Negative, AscI site, reverse
Pr3	atatgcggccgctaaggaggaacatattttatgaaaggttttgc	To amplify isopropanol producing operon *sadh-hydG* from *C. beijerinckii* and insert into pMTL JH16 to construct pMTL-JH16-*sadh-hydG* B593, NotI site, forward
Pr4	atatgctagcttatttatcacctctgcaaccacagcc	To amplify isopropanol producing operon *sadh-hydG* from *C. beijerinckii* and insert into pMTL JH16 to construct pMTL-JH16-*sadh-hydG* B593, NheI site, reverse
Pr5	tagcacaattgtatttggacttctttaaataaaaacatgg	To confirm pSOL1 integration at 5', located on *CA_C0026* on chromosome, forward
Pr6	gaacgcagtaaggtttacaagtc	To confirm pSOL1 integration at 5', located on *CA_P0177* on pSOL1, reverse

Pr7	tgaatccatacgagacgtaatacaa	To confirm pSOL1 integration at 3', located on *CA_P0173* on pSOL1, forward
Pr8	gttgatcctgctatgggtactt	To confirm pSOL1 integration at 3', located on *hydA (CA_C0028)* on chromosome, reverse
Pr9	gcaggagatgctgacgtaataa	To confirm *sadh-hydG* integration into chromosome under *thlA* promoter, located on *thlA*, forward
Pr10	ccatgaagaggtactggcaataa	To confirm *sadh-hydG* integration into chromosome under *thlA* promoter, located on *atpB*

[A)] **Underline indicates restriction sites**

Supplementary Table 2. List of mutations identified in CAB2018 strain

Gene	Position	Length	Type	Nucleotide change	AA substitution	Function	No. of mRNA molecules/cell in acidogenesis A)	No. of mRNA molecules/cell in solventogenesis A)
CA_C1879	2217909	1	SNP	A→G	Ile260Val	Hypothetical protein	0.068	0.000
CA_C2134	2415895	1	SNP	G→A	Gly241Ser	GTP-dependent nucleic acid binding protein EngD	2.293	2.852
CA_C2912 araN	3237960	11	Deletion	delATGTATATGAA	NP_349514.1:p.Met377fs	Sugar binding periplasmic protein	0.915	0.667
CA_C3406	3780000	2	Insertion	171_172dupTG	NP_349996.1:p.Glu58fs	Transcriptional regulator	0.111	0.098

A) Quantitated mRNA under acidogenesis or solventogenesis in *C. acetobutylicum* ATCC 824. The minimum number of mRNA molecules per cell detected was around 0.06 while the maximum number was around 80 in this study[5].

Supplementary Note 1 | Transformation procedure

C. acetobutylicum ATCC 824 and derived strains were electroporated as previously described [6], in vivo methylation of plasmid DNA was carried by transformation in TOP10 harbouring pAN2 (Supplementary Table 1) with selection by the use of 10 µg/mL of tetracycline and by the use of appropriate antibiotic specific for the plasmid to methylate (chloramphenicol 30 µg/mL for all *catP*-carrying plasmids) prior to transformation of any *C. acetobutylicum* strains[1]. After transformation and recovery in liquid 2×YTG (pH 5.2), cells were plated on CGM agar supplemented with the appropriate antibiotics (erythromycin at 40 µg/ml and thiamphenicol at 20 µg/ml, respectively) and with uracil at 20 µg/ml in the case of *pyrE* negative mutant. Transformants selected on media containing antibiotics were subcultured twice on the same media for purification. The final clones screened by PCR were grown in synthetic media (GMM containing ammonium acetate) for product profile verification and spore storage.

Supplementary Note 2 | Construction of CAB2018 strain

Plasmid pMTL-pSOL1-int (Fig. 1, Supplementary Table 1, and Supplementary Figure 1) was constructed as described in the main manuscript. The vector was transformed into *pyrE* engineered strain [3]. Mutants having a clean integration of pSOL1, resulting from four crossing over events (Fig.1a), were directly obtained by spreading 10^9 of pMTL-pSOL1-int plasmid containing cells on CBM agar supplemented with erythromycin (*ermB*). Less than 5 colonies were routinely obtained.
All the resultant clones were replicated on CBM agar supplemented with erythromycin and 5FC to select for the clones cured of the original ACE plasmid, as well as the hybrid ACE plasmid with two origins of replication that was generated after the first double crossing over event. Both 5′ and 3′ integrations of pSOL1 were checked by PCR (Supplementary Figure 2). All the clones analyzed had the PCR profile corresponding to an integration of pSOL1. One of the Erm^R Tm^S $5FC^S$ clones was named CAB2018.

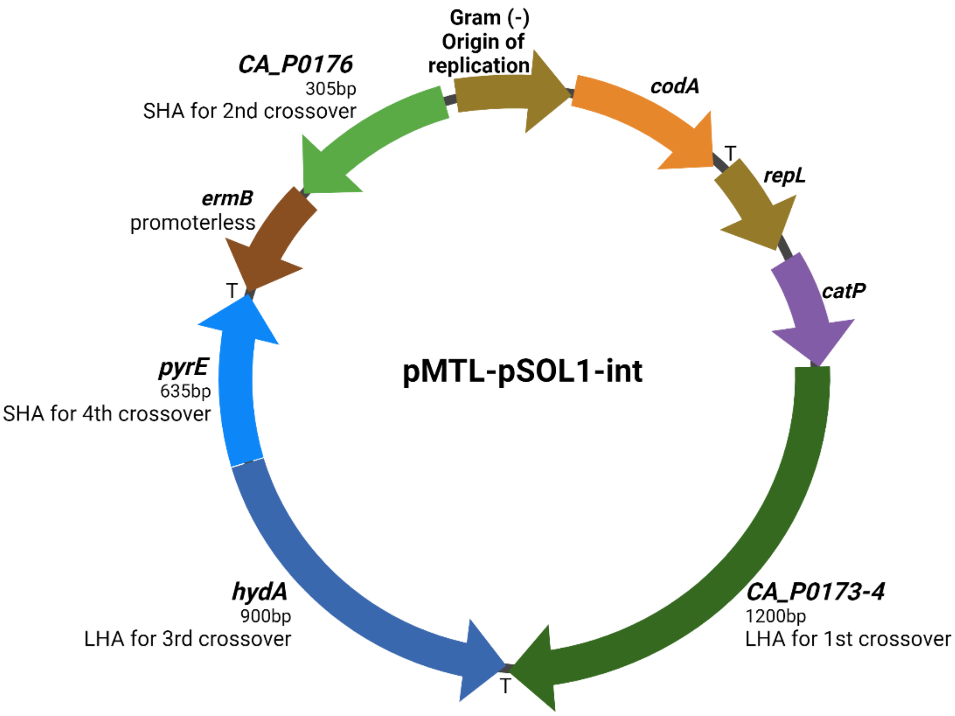

Supplementary Figure 1. Physical map of pMTL-pSOL1-int

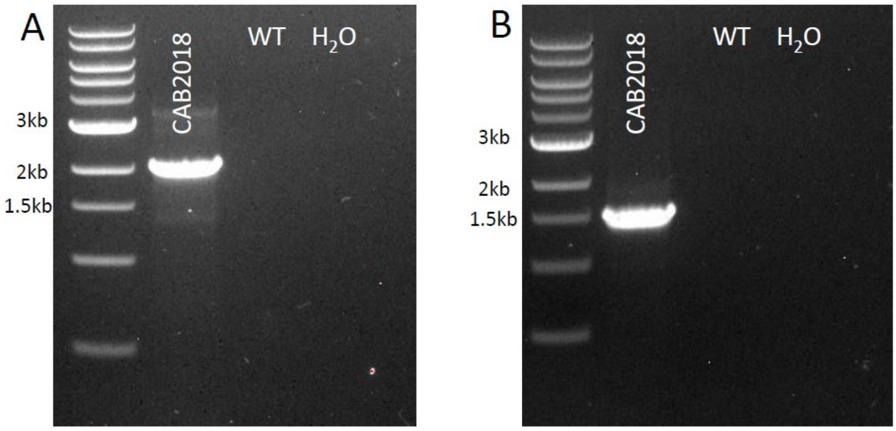

Supplementary Figure 2. pSOL1 integration verification by PCRs in CAB2018. (a) 5′ integration verification with primer pair Pr5 located on *CA_C0026* on chromosome and Pr6 located on *CA_P0177* on pSOL1. Expected amplicon size: CAB2018, 2.1kb; WT, no amplicon. NEB 1 kb DNA Ladder was loaded on the left. (b) 3′ integration verification with primer pair Pr7 located on pSOL1 and Pr8 located on chromosome. Expected amplicon sizes: CAB2018, 1.5 kb; WT, no amplicon.

Supplementary Note 3 | Construction of CAB2019, CAB2020 and CAB2021 strains

CAB2019

CAB2018 harbours *ermB* (erythromycin resistance gene, used for double-crossover selection during pSOL1 integration into chromosome and to be used for double-crossover selection later on to use its high selection efficiency again in this organism) and to remove *ermB* it was transformed with pMTL-JH12 derived vector pMTL-pSOL1-*pyrE*-Negative, which was constructed as described in the main manuscript. Transformants were plated on CGM supplemented with 20 µg/mL uracil and 15 µg/mL thiamphenicol for the plasmid-borne resistance marker *catP* selection. The clones were subcultured twice on the same media then streaked on CBM (normally lacking in uracil)[7] supplemented with 20 µg/mL uracil and 1 mg/ml 5-fluoroorotic acid (FOA) for counterselection. The cells resistant to FOA were streaked onto CBM agar supplemented with uracil and erythromycin to confirm deletion of *ermB*. Accordingly, the erythromycin sensitive clones were verified by PCR and subsequently streaked onto CBM agar deficient in uracil to confirm uracil auxotrophy due to non-functional foreshortened *pyrE* as well as on uracil supplemented CBM containing thiamphenicol to ensure loss of the integration vector. The resultant Erm^S Tm^S FOA^R strain was named CAB2019.

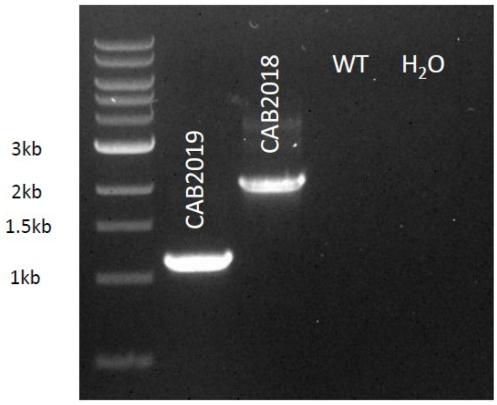

Supplementary Figure 3. PCR validation of CAB2019 strain
The *ermB* excised and *pyrE* deleted CAB2019 strain was verified by PCR. Primers used: Pr5 located on *CA_C0026* on chromosome and Pr6 located on *CA_P0177* on pSOL1. Expected amplicon sizes: CAB2019 strain, 1 kb; CAB2018 strain, 2.1 kb; WT, no amplicon. NEB 1 kb DNA Ladder was loaded on the left.

CAB2020

To restore a full-length functional *pyrE,* CAB2019 was transformed with pMTL-pSOL1-*pyrE*-Positive by electroporation and plated on CBM supplemented with thiamphenicol and uracil. After subculturing on the same medium, the transformants were transferred to CBM lacking uracil to select uracil prototrophic cells. The cells grown on CBM were purified for PCR screening. The clones showing correct PCR profiles were streaked on CGM supplemented with FOA. The clones with sensitivity to FOA and which were capable of growing on CBM without uracil supplementation were streaked on CBM containing thiamphenicol to ensure loss of the integration vector. The resultant Erm^S Tm^S FOA^S strain was named CAB2020.

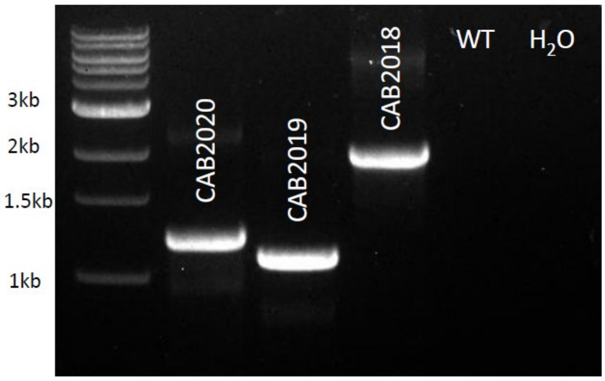

Supplementary Figure 4. PCR validation of CAB2020 strain. The antibiotic resistance marker (encoded by *ermB*) excised and *pyrE* restored CAB2020 strain was verified by PCR. Primers used: Pr5 located on *CA_C0026* on chromosome and Pr6 located on *CA_P0177* on pSOL1. Expected amplicon size: CAB2020 strain, 1.3 kb; CAB2019 strain, 1 kb; CAB2018 strain, 2.1 kb; WT, no amplicon. NEB 1 kb DNA Ladder was loaded on the left.

CAB2021

CAB2020 strain was transformed with pMTL-JH16-*sadh-hydG* B593, which was constructed as described in the main manuscript. After recovery in 2×YTG, cells were plated on CGM supplemented with thiamphenicol and subcultured on the same medium. Purified clones were replicated on CGM supplemented with erythromycin and subcultured on the same medium to select double-crossover clones as *ermB* gene was designed to be under *thlA* promoter after integration. Afterwards, Erm^R clones were screened by PCR and thiamphenicol sensitivity. The resultant Erm^R Tm^S recombinant strain was named CAB2021.

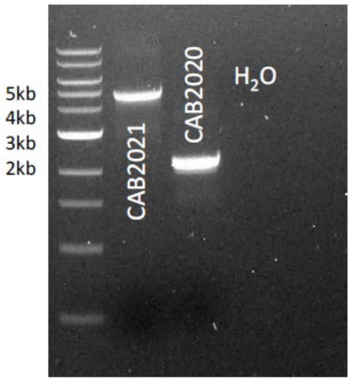

Supplementary Figure 5. PCR validation of CAB2021 strain
Isopropanol producing (by *sadh-hydG* integration) pSOL1 integrated CAB2021 strain was verified by PCR. Primers used: Pr9 on *thlA* (*CA_C2873*) and Pr10 located on *atpB* (*CA_C2871*). Expected amplicon sizes: CAB2021 strain, 5 kb; CAB2020 strain, 2.3 kb. NEB 1 kb DNA Ladder was loaded on the left.

Supplementary Note 4 | Specific growth rate, products profile in serum bottles and sporulation ability of the wild type and CAB2018 strains.

Supplementary Table 3. Specific growth rate in serum bottles of the wild type and CAB2018 strains

In GMM containing glucose and acetate under batch condition without pH regulation, CAB2018 showed a significant decrease in specific growth rate compared to wild type strain. Data are shown as mean ± SD from biological replicates (n = 3)

	**Wild type**	**CAB2018**
Specific growth rate (h^{-1})	0.36 $\pm$ 0.05	0.26 $\pm$ 0.01

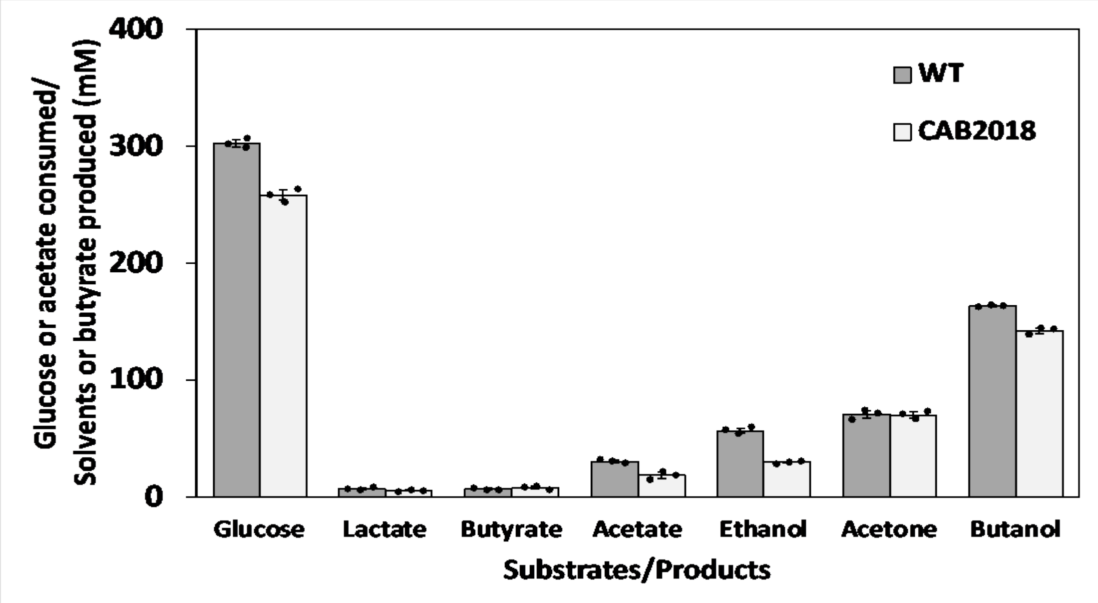

Supplementary Figure 6. Products profile in serum bottles of the wild type and CAB2018 strains

In minimal media (GMM) containing glucose and acetate under batch condition without pH regulation, CAB2018 showed slightly decreased glucose and decreased acetate consumption and consequently a slight decrease in both butanol and ethanol production. Data are shown as mean ± SD from biological replicates (n = 3)

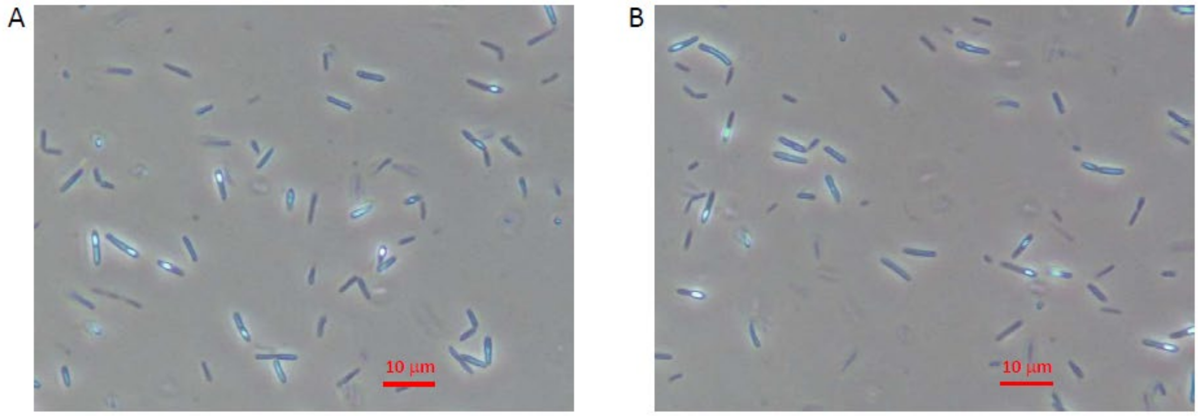

Supplementary Figure 7. Sporulation of CAB2018
Microscopic images of spore formation of *C. acetobutylicum* strains: (a) Wild type, (b) CAB2018. As it is known that the processes of solvent production and sporulation are linked, and degenerated *C. acetobutylicum* can no longer sporulate [8, 9], the sporulation ability of the pSOL1 integrated strain was examined and confirmed by microscopy.

Supplementary Note 5 | Products profile in serum bottles of the wild type, CAB2020 and CAB2021 strains.

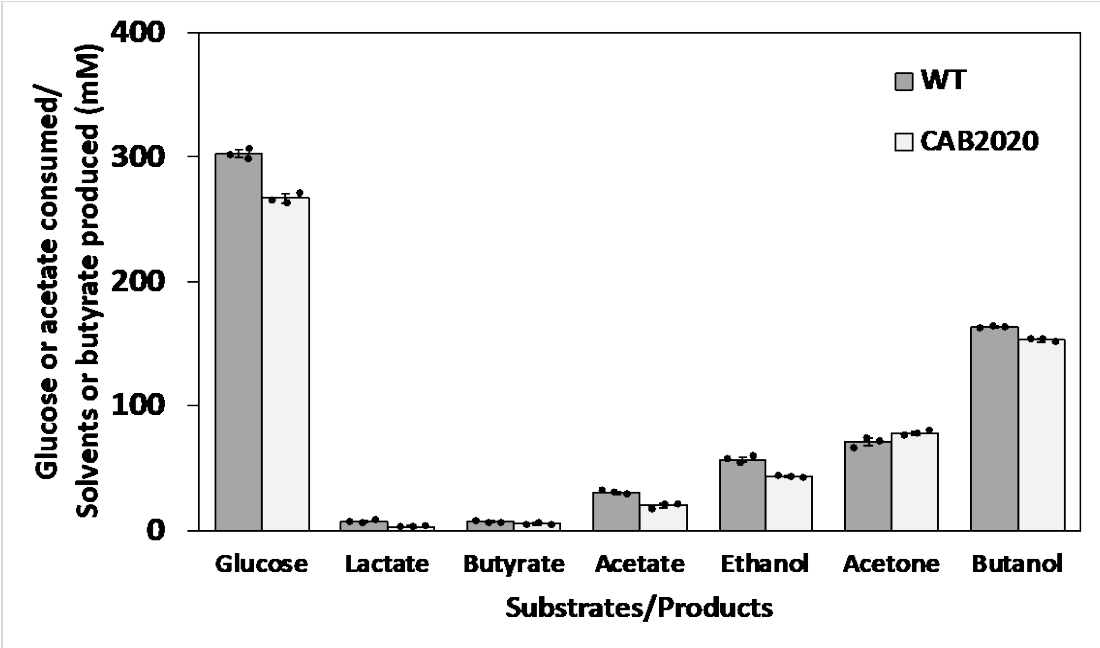

Supplementary Figure 8. Products profile in serum bottles of the wild type and CAB2020 strains
The product profile of CAB2020 in GMM under batch conditions without pH regulation showed slightly decreased glucose consumption and consequently a slight decrease in both butanol and ethanol production compared to WT. This profile is similar to the parental strain CAB2018. Data are shown as mean ± SD from biological replicates (n = 3).

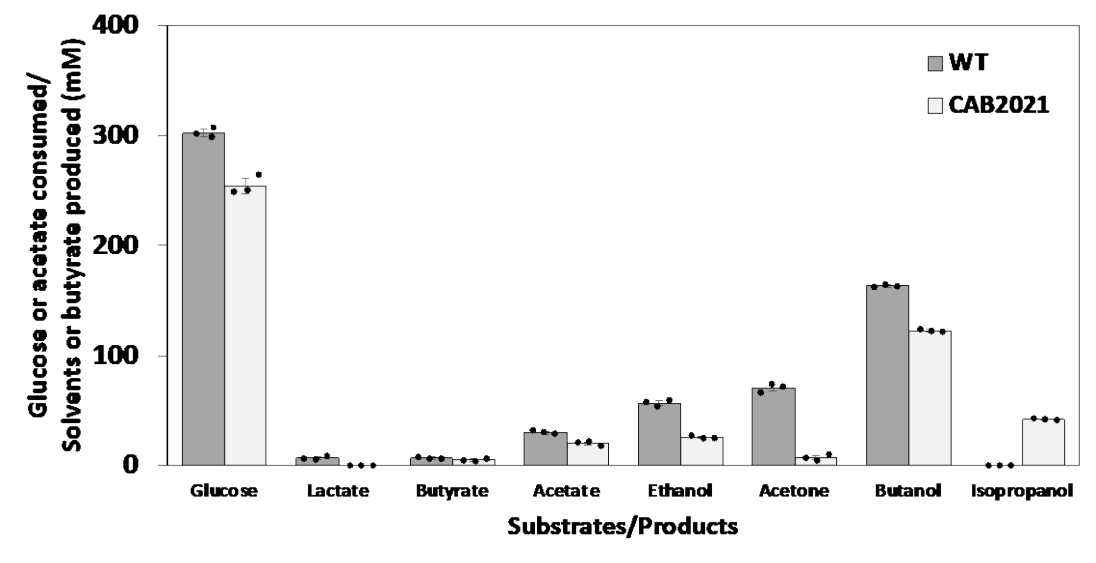

Supplementary Figure 9. Products profile in serum bottles of the wild type and CAB2021 strains

The product profile of CAB2021 in GMM under batch condition without pH regulation showed slightly decreased glucose consumption, similar to its parental strains CAB2018, CAB2020. This strain showed significant production of isopropanol and very low production of acetone due to its conversion to isopropanol. Data are shown as mean ± SD from biological replicates (n = 3)

Supplementary Note 6 | Analysis of pSOL1 loss in wild type and CAB2019 strains.

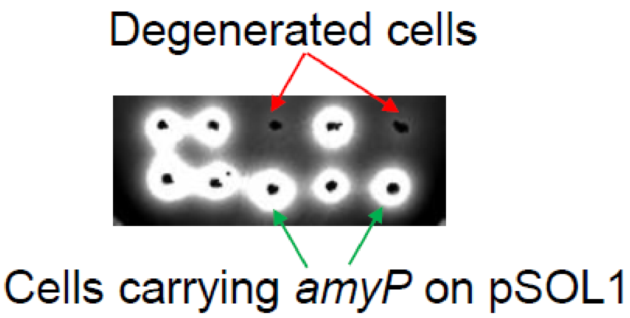

Supplementary Figure 10. Starch based assay to analyze loss of pSOL1.

Halo formations by amylolytic activity of gene product of *amyP*, located on pSOL1, were monitored to determine the degeneration rate[4, 10]. As AmyP is solely able to form halo without catabolic repression, assays on starch plates supplemented with limited glucose were implemented during fermentation (Main text Figure 1). In WT fermentation, once degeneration was detected, the rate at which the population of no halo forming cells, indicative of pSOL1 loss, increased gradually. At the same time, butyrate production increased and butanol production decreased within 30 days, all tested cells showed no halo formation (less than 1% of average until day 20) and no butanol production (Fig. 1 B and C). On the other hand, as CAB2019 strain carried pSOL1 including *amyP* in pSOL1 integrated into chromosome, no cells unable to form halo were observed throughout the entire fermentation period with the accompanying stable butanol production (42 days).

Supplementary Note 7 | Genome Sequencing of CAB2018 strain**.**

Genomic DNA was isolated from overnight cultures of the *C. acetobutylicum* strain to be sequenced using phenol-chloroform extraction and stored in Tris-HCl buffer (10 mM, pH 7.8). Genomic DNA was sequenced using the Illumina MiSeq platform (DeepSeq, Nottingham, UK) using 500 bp V2 SBS chemistry. Analysis of the generated reads and identification of single nucleotide polymorphisms, insertions and deletions was performed using the Basic Variant Detection tool within CLC Genomics Workbench version 22.0.1 (Qiagen) by mapping the trimmed Illumina paired-end reads to the reference sequences NC_003030 (chromosome) and NC_001988 (pSOL1) modified according to Ehsaan *et al.*[4]. Variants were detected with minimum coverage and frequency of 75 and 70%, respectively, with base quality filter settings of neighbourhood radius = 5, minimum central quality = 20 and minimum neighbourhood quality = 15, applied.
The sequencing results are available as a supplementary file.

Supplementary Note 8 Comparison of the IBE production by the natural or engineered Clostridia in continuous culture

Supplementary Table 4. Comparison of the IBE production by Clostridia in continuous culture

Strain	Metabolic Engineering Strategy	Substrate/ Medium	Fermentation Type	IBE Yield ($g \cdot g^{-1}$)	IBE Titre ($g \cdot L^{-1}$)	IBE Productivity ($g \cdot L^{-1} \cdot h^{-1}$)	Reference
***C. beijerinckii* NRRL B593**	WT	Sucrose/ Complex medium	Continuous Chemostat	NA	3.1	0.372	Ahmed et al.[11]
***C.acetobutylicum* ATCC 824 (*sadh*)**	*sadh* at the *pyrE* locus	Glucose/ Mineral medium	Continuous Chemostat	0.28	5.2	0.39	Bankar et al.[12]
***C. beijerinckii* DSM 6423**	WT	Glucose/ Complex medium	Continuous Two stage	0.32	7.6	0.11	Survase et al.[13]
***C. beijerinckii* DSM 6423**	WT	Glucose/ Complex medium	Continuous Immobilized	0.29	9.2	5.52	Survase et al.[13]
***C. beijerinckii* DSM 6423**	WT	Glucose/ Complex medium	Continuous Immobilized	0.3	13.5	2.5	Carrié et al.[14]
C. beijerinckii optinoii	WT	Glucose/ Mineral medium	Continuous Immobilized	0.26	12.7	1.08	Yang et al.[15]
***C. beijerinckii* NRRL B593**	WT	Glucose/ Complex medium	Continuous Immobilized	0.32	5.5	4.0	Krouwel et al.[16]
***C. beijerinckii* DSM 2152**	WT	Glucose/Complex medium	Continuous Extractive Gas Striping	0.19	NA	0.63	Groot et al.[17]
***C.acetobutylicum* ATCC 824 (*sadh*)**	*sadh* at the *pyrE* locus	Glucose/ Mineral medium	Continuous Membrane bioreactor	0.23	10.6	7.0	Survase et al.[18]
***C.acetobutylicum* CAB2021**	pSOL1 integrated at the *pyrE* locus, *sadh-hydG* at the *thlA* locus	Glucose/ Mineral medium	Continuous Membrane bioreactor	0.31	15.1	15.5	This study

Supplementary Note 9 | Schematic representation of the natural organization of the genes used for engineering in this study

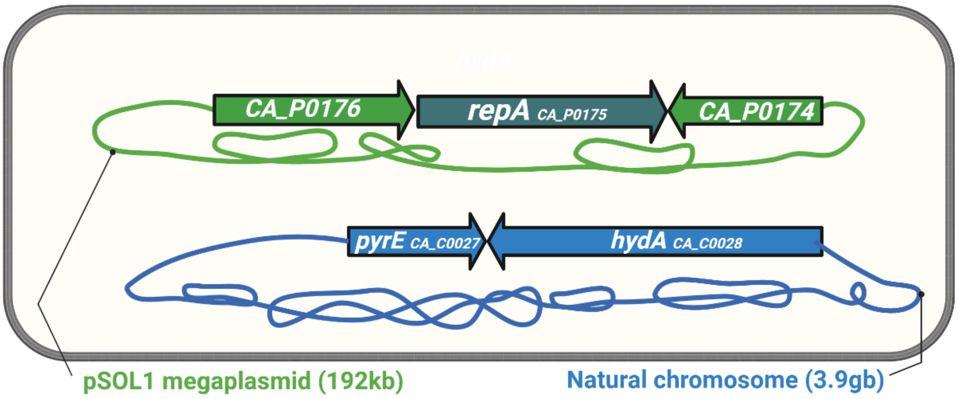

Supplementary Figure 11. Natural organization of the genes used for engineering in this study

Supplementary Note 10 | Central metabolism of *Clostridium acetobutylicum*

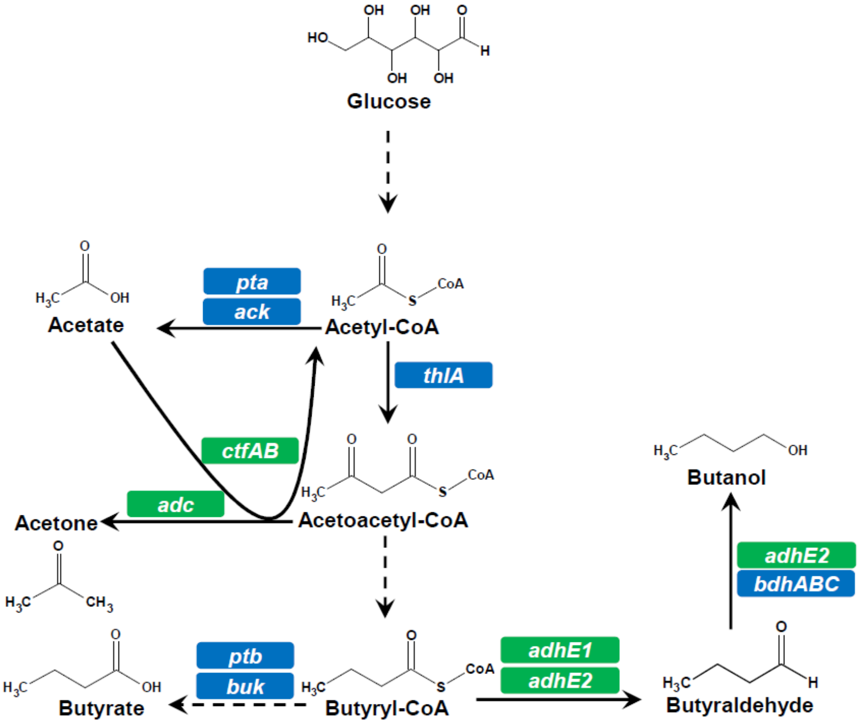

Supplementary Figure 12. Simplified central metabolism of *Clostridium acetobutylicum*

Blue box indicates genes on chromosome whereas green box indicates genes on pSOL1 megaplasmid. Abbreviations: *ack*, acetate kinase; *adc*, acetoacetate decarboxylase; *adhE1*, aldehyde dehydrogenase; *adhE2*, bifunctional aldehyde/alcohol dehydrogenase; *bdhABC*, butanol dehydrogenase; *buk*, butyrate kinase; *ctfAB*, CoA-transferase; *pta*, phosphotransacetylase; *ptb*, phosphotransbutyrylase; *thl*, thiolase.

References

1. Heap, J.T., Pennington, O.J., Cartman, S.T., Carter, G.P. & Minton, N.P. The ClosTron: a universal gene knock-out system for the genus Clostridium. *J Microbiol Methods* **70**, 452-464 (2007).
2. Mermelstein, L.D. & Papoutsakis, E.T. In vivo methylation in Escherichia coli by the Bacillus subtilis phage phi 3T I methyltransferase to protect plasmids from restriction upon transformation of Clostridium acetobutylicum ATCC 824. *Appl Environ Microbiol* **59**, 1077-1081 (1993).
3. Heap, J.T. et al. Integration of DNA into bacterial chromosomes from plasmids without a counter-selection marker. *Nucleic Acids Res* **40** (2012).
4. Ehsaan, M. et al. Mutant generation by allelic exchange and genome resequencing of the biobutanol organism Clostridium acetobutylicum ATCC 824. *Biotechnol Biofuels* **9**, 4 (2016).
5. Yoo, M. et al. A quantitative system-scale characterization of the metabolism of Clostridium acetobutylicum. *mBio* **6**, e01808-01815 (2015).
6. Mermelstein, L.D., Welker, N.E., Bennett, G.N. & Papoutsakis, E.T. Expression of cloned homologous fermentative genes in Clostridium acetobutylicum ATCC 824. *Biotechnology (N Y)* **10**, 190-195 (1992).
7. O'Brien, R.W. & Morris, J.G. Oxygen and the growth and metabolism of Clostridium acetobutylicum. *J Gen Microbiol* **68**, 307-318 (1971).
8. Cornillot, E., Nair, R.V., Papoutsakis, E.T. & Soucaille, P. The genes for butanol and acetone formation in Clostridium acetobutylicum ATCC 824 reside on a large plasmid whose loss leads to degeneration of the strain. *J Bacteriol* **179**, 5442-5447 (1997).
9. Al-Hinai, M.A., Jones, S.W. & Papoutsakis, E.T. The Clostridium sporulation programs: diversity and preservation of endospore differentiation. *Microbiol Mol Biol Rev* **79**, 19-37 (2015).
10. Sabathe, F., Croux, C., Cornillot, E. & Soucaille, P. amyP, a reporter gene to study strain degeneration in Clostridium acetobutylicum ATCC 824. *FEMS Microbiol Lett* **210**, 93-98 (2002).
11. Ahmed, I., Ross, R., Mathur, V. & Chesbro, W. Growth rate dependence of solventogenesis and solvents produced by Clostridium beijerinckii. *Appl Microbiol Biot* **28**, 182-187 (1988).
12. Bankar, S.B., Jurgens, G., Survase, S.A., Ojamo, H. & Granstrom, T. Genetic engineering of Clostridium acetobutylicum to enhance isopropanol-butanol-ethanol production with an integrated DNA-technology approach. *Renew Energy* **83**, 1076-1083 (2015).
13. Survase, S.A., Jurgens, G., van Heiningen, A. & Granstrom, T. Continuous production of isopropanol and butanol using Clostridium beijerinckii DSM 6423. *Appl Microbiol Biotechnol* **91**, 1305-1313 (2011).
14. Carrié, M., Velly, H., Ben-Chaabane, F. & Gabelle, J.C. Modeling fixed bed bioreactors for isopropanol and butanol production using DSM 6423 immobilized on polyurethane foams. *Biochem Eng J* **180** (2022).
15. Yang, Y., Hoogewind, A., Moon, Y.H. & Day, D. Production of butanol and isopropanol with an immobilized Clostridium. *Bioproc Biosyst Eng* **39**, 421-428 (2016).
16. Krouwel, P., Groot, W. & Kossen, N. Continuos IBE fermentation by immobilized growing Clostridium beijerinckii cells in a stirred-tank fermentor. *Biotechnology and bioengineering* **25**, 281-299 (1983).

17. Groot, W., Van der Lans, R. & Luyben, K.C.A. Batch and continuous butanol fermentations with free cells: integration with product recovery by gas-stripping. *Appl Microbiol Biot* **32**, 305-308 (1989).
18. Survase, S.A. et al. Membrane assisted continuous production of solvents with integrated solvent removal using liquid-liquid extraction. *Bioresour Technol* **280**, 378-386 (2019).
